# Public health in community pharmacy: A systematic review of pharmacist and consumer views

**DOI:** 10.1186/1471-2458-11-582

**Published:** 2011-07-21

**Authors:** Claire E Eades, Jill S Ferguson, Ronan E O'Carroll

**Affiliations:** 1 Department of Psychology, University of Stirling, Stirling, FK9 4LA, Scotland, UK; 2 NHS Forth Valley Pharmacy Services, Eurohouse, Wellgreen Place, Stirling, FK8 2DJ, Scotland, UK

## Abstract

**Background:**

The increasing involvement of pharmacists in public health will require changes in the behaviour of both pharmacists and the general public. A great deal of research has shown that attitudes and beliefs are important determinants of behaviour. This review aims to examine the beliefs and attitudes of pharmacists and consumers towards pharmaceutical public health in order to inform how best to support and improve this service.

**Methods:**

Five electronic databases were searched for articles published in English between 2001 and 2010. Titles and abstracts were screened by one researcher according to the inclusion criteria. Papers were included if they assessed pharmacy staff or consumer attitudes towards pharmaceutical public health. Full papers identified for inclusion were assessed by a second researcher and data were extracted by one researcher.

**Results:**

From the 5628 papers identified, 63 studies in 67 papers were included. *Pharmacy staff: *Most pharmacists viewed public health services as important and part of their role but secondary to medicine related roles. Pharmacists' confidence in providing public health services was on the whole average to low. Time was consistently identified as a barrier to providing public health services. Lack of an adequate counselling space, lack of demand and expectation of a negative reaction from customers were also reported by some pharmacists as barriers. A need for further training was identified in relation to a number of public health services. *Consumers: *Most pharmacy users had never been offered public health services by their pharmacist and did not expect to be offered. Consumers viewed pharmacists as appropriate providers of public health advice but had mixed views on the pharmacists' ability to do this. Satisfaction was found to be high in those that had experienced pharmaceutical public health

**Conclusions:**

There has been little change in customer and pharmacist attitudes since reviews conducted nearly 10 years previously. In order to improve the public health services provided in community pharmacy, training must aim to increase pharmacists' confidence in providing these services. Confident, well trained pharmacists should be able to offer public health service more proactively which is likely to have a positive impact on customer attitudes and health.

## Background

Promotion of healthy lifestyles is one of the five core roles of a pharmacist, as defined by the Royal Pharmaceutical Society of Great Britain, (RPSGB) [1]. Although pharmacists have always had some involvement in health improvement, the focus on this aspect has greatly increased over recent years [2]. This changing role was formalised by the introduction of the new pharmacy contract in 2005 in England and Wales and 2006 in Scotland which outlined the public health service pharmacists would be required to provide. These services include provision of advice on healthy living and self care and involvement in health promotion campaigns in Scotland, England and Wales with the additional requirement to provide a smoking cessation and sexual health service in Scotland [3,4].

Community pharmacy holds a number of benefits as a setting for public health activities. With extended opening hours and no appointment needed for advice, community pharmacy can be more accessible than other settings. An estimated 600,000 people visit community pharmacies in Scotland every day and approximately 94% of the Scottish population visit a community pharmacy at least once in a year [5]. This gives community pharmacies access to a range of individuals in both good and poor health, and to those that may not have contact with any other health professionals. Reviews of evidence assessing public health initiatives in community pharmacy have confirmed the potential of pharmacy in this area and suggest that pharmacists can indeed make a positive contribution to public health [6,7].

Although there is clear potential for pharmacy to contribute in a unique way to public health, changes in the behaviour of both pharmacists and pharmacy customers are likely to be required for the service to be successful. Pharmacists must accept their role in public health and make the necessary changes in behaviour to carry out the service. Similarly, the general public must accept pharmacists as providers of public health services and be willing to seek advice on some health issues from pharmacists rather than other sources.

The factors that affect and predict behaviour have been the subject of a great deal of research. The theory of planned behaviour (TPB) is a model that has been widely used to predict and change behaviour across a range of settings [8]. The model states that voluntary behaviours are largely predicted by our intentions regarding the behaviour. Intentions are in turn determined by our attitude towards the behaviour (our judgement of whether the behaviour is a good thing to do), subjective norms (our judgement of what important others think of the behaviour), and perceived behavioural control (our expectation of how successful we will be in carrying out the behaviour). A review by Sutton found that on average the TPB predicted between 40 and 50% of the variance in intention and between 19 and 38% of the variance in behaviour [9]. While theories such as the TPB cannot entirely predict behaviour, these findings demonstrate the important role of beliefs in understanding behaviour.

Therefore, in order to understand and assist the behaviour changes associated with providing a public health service in community pharmacy, it is important to establish the beliefs of the general public and pharmacists regarding this role. Three systematic reviews have previously been carried out in this area. One assessed pharmacist views and another general public views towards various public health services [10,11]. The third reviewed papers on the provision of emergency hormonal contraception (EHC) in pharmacy and included public and pharmacist views [12]. The review of pharmacists' perceptions of public health covered literature published up to 2001 and found that although pharmacists valued the health improvement role they were more comfortable with medicine related health improvement work [10]. The review also found that pharmacists had concerns about being intrusive and believed they needed more support to provide public health services. Training was found to positively affect pharmacists' attitudes and behaviours in relation to health promotion [10].

The review on consumer views covered literature up to 2002 and found that pharmacists were perceived as 'drug experts' rather than experts on health and illness. Although consumers were generally satisfied with health advice given by pharmacists, they primarily used pharmacies for dispensing prescriptions and buying over the counter medication [11]. The final review summarised literature on the provision of EHC in pharmacy up to the end of 2004. The review reported that the service was largely viewed positively by both pharmacists and service users but that some concerns were raised by consumers regarding privacy [12].

Since these reviews were conducted, the introduction of the new pharmacy contract has brought about a great deal of change in community pharmacies. In order to continue to improve the public health service provided in community pharmacies, up to date information is needed regarding the beliefs and attitudes of pharmacists and consumers towards pharmaceutical public health. Beliefs about the public health role may or may not be similar to those found in the previous review. Establishing current views would allow potential barriers to the public health service to be established and appropriately tackled. The objective of this review is to summarise and evaluate quantitative and qualitative evidence published since the previous reviews were conducted on the beliefs and attitudes of pharmacists and consumers towards pharmaceutical public health.

## Methods

The electronic databases MEDLINE, EMBASE, PsycINFO, CINAHL and Dissertation Abstracts International were searched for articles published in English from February 2001 to February 2010. The following combination of search terms was used with each database: (pharm* or pharmacy staff or community pharmacy or consumer or public or customer) and (attitud* or belie* or perce* or knowledge or view or opinion) and (public health or health improvement or health promotion or self care or self management or smoking cessation or sexual health or prevent* or diet or healthy diet or healthy eating or exercise or physical activity or weight or health education or chlamydia testing or emergency contraception or alcohol or needle exchange or methadone or injecting equipment or drug misuse).

Titles and abstracts were screened against the inclusion criteria outlined in table 1. Full text papers were retrieved for studies considered relevant and for those with titles and abstracts that contained insufficient information to allow judgement of relevance. The full text papers were assessed against the inclusion criteria by one researcher and those identified as relevant were checked again by a second researcher. Data were extracted from included studies using a data extraction form based on the example provided by the Centre for Reviews and Dissemination [13]. In order to assess methodological quality, studies were assessed against the checklist outlined by Crombie which is suitable for use with descriptive surveys [14]. The methodological quality of qualitative studies was assessed against the Critical Appraisal Skills Programme checklist for qualitative studies [15].

**Table 1:** Criteria for inclusion of studies in the review

Population	Community pharmacists, community pharmacy support staff, pharmacy customers and members of the general public.
**Phenomenon of Interest**	The phenomenon of interest was the attitudes and beliefs of the general public and community pharmacists towards the public health role of community pharmacists. The activities included in the scope of pharmacy public health services in this review include individual and community efforts to promote health, prevent and detect health problems and promote self care in individuals with long term health conditions.

**Primary Outcome Measures**	The outcome measures of interest included but were not restricted to the following: • Pharmacists' and general public's beliefs about the skills and knowledge of pharmacists in providing public health • Pharmacists' and general public's beliefs about the suitability of pharmacists and community pharmacy for providing public health services. • Pharmacists' and general public's beliefs about the most appropriate provider of public health services. • Pharmacists' beliefs about their confidence in carrying out public health services. • Pharmacists' beliefs about training needed for carrying out public health services. • General public's views about their experiences of public health services in community pharmacy. • General public's use of community pharmacies and knowledge of public health services provided in pharmacies.

**Types of Studies**	Quantitative cross sectional surveys and qualitative studies. Studies were included if they reported one or more of the outcomes detailed above.

## Results

### Literature Search

**Figure 1:**
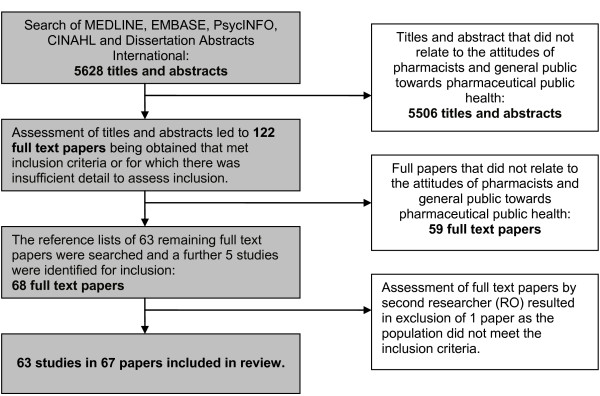
**Flow diagram of searches and inclusion assessment of studies**.

A total of 5628 abstracts were reviewed and 122 full text papers were assessed against the inclusion criteria outlined in Table 1. A second researcher assessed the 71 papers shortlisted for inclusion and 63 studies published in 67 papers were included for review. Figure 1 shows the flow of studies identified by the searches.

### Description of Included Studies

The characteristics of the studies included in the review are presented in additional file 1. The majority of studies assessed the views of pharmacists (n = 29), support staff (n = 3) or both (n = 1). Three studies investigated both pharmacist and general public views and the remaining studies assessed the views of the general public or pharmacy customers (n = 27). The most common topics investigated were sexual health services (n = 17), smoking cessation (n = 14), general health promotion/screening (n = 12), and services for drug misusers (n = 10). The majority of studies were carried out in Europe (n = 31) and North America (n = 23). The most commonly employed methodology was surveys (n = 50). Eight studies used structured or semi-structured interviews, two used focus groups and two studies used both focus group and survey methods. Table 2 outlines the country of publication of papers included in the review sorted by topic area. It shows the proportion of UK and non-UK papers published after the introduction of the new pharmacy contract in the UK (2006 to 2010).

**Table 2:** Details of country and date of publication of included papers by topic area

Topic	Total No. of studies	No. of UK studies published 2006-2010 (% total UK studies)	No. of non UK published 2006-2010 (% total non UK studies)	Country Published (No.)
Smoking Cessation	13	n/a	7 (54%)	USA (7), Canada (2), Finland (1), Turkey (1), Australia (1), Thailand (1).

EHC	12	1 (33%)	4 (44%)	USA (4), UK (3), Sweden (2), Canada (1), Various Europe (1), Australia (1).

Health Promotion and Screening	12	2 (66%)	6 (66%)	UK (3), Sweden (2), Nigeria (2), Australia (2), Moldova (1), Malaysia (1), USA (1).

Drug Misuse	12	6 (66%)	2 (66%)	UK (9), USA (1), Vietnam (1), Estonia (1).

Chlamydia	5	4 (100%)	0	UK (4), The Netherlands (1).

Osteoporosis Screening	3	1 (100%)	0	USA (2), UK (1).

Type 2 Diabetes	2	n/a	2 (100%)	United Arab Emirates (1), USA (1).

Weight Management	2	1 (100%)	0	USA (1), UK (1).

Alcohol consumption	1	n/a	1 (100%)	New Zealand (1).

Asthma Management	1	n/a	1 (100%)	Finland (1).

Total	63	15 (71%)	23 (55%)	United Kingdom (21), Non-UK Europe (10), North America (20), Asia (5), Oceania (5), Africa (2).

### Quality of Included Studies

Quality varied across the studies included. The quality of reporting was often poor with 16 studies not reporting any information on the age of participants [16-35], 8 not reporting age or gender [36-43] and 2 not reporting gender [44,45]. Fifteen studies did not report response rates [17,25,29,43,46-56] and two only reported the response rates for part of the sample [57,58]. Only three studies followed up a sample of non-responders [59-61]. Response rates where reported were generally average to good with the majority (71%) achieving response rates of 50% and over. The way participants were recruited was not clearly reported in one study [50] and the results were not adequately explained in another [62]. In the latter case, the names of themes arising from the analysis of interviews were stated with little explanation of the direction of opinion of pharmacists in relation to these themes. The majority of studies included in the review employed convenience sampling (n = 29), 5 used purposive sampling [41,56,62-64] and only 13 used random sampling methods [16,18,32-38,47,50,58,65-68]. Of the 12 studies included that used qualitative methodologies only one employed respondent validation [62] or made a statement of how the personal characteristics of the researchers may have influenced analysis [69]. Methods and analysis were not adequately described in one study [43], data was not transcribed verbatim in another study [70] and multiple coding was not used in two further studies [41,51].

### Pharmacy Staff

The attitudes and beliefs of pharmacists and pharmacy staff investigated in the papers included in this review related to four main topics: perceptions of role, competence/confidence, barriers and training.

#### Perceptions of Role

The majority of participants in a survey in Scotland agreed (63%) or strongly agreed (16%) that public health is important to their practice and a little over half agreed (48%) or strongly agreed (8%) that they were public health practitioners [21]. A survey in Nigeria also reported that the majority of participants (94%) thought it was acceptable for pharmacists to be involved in health promotion activities [71]. Pharmacists and support staff taking part in focus groups in Sweden on the whole welcomed their role as a health promoter [56]. However, it was noted that not all participants felt this way and preferred to develop activities in areas in which they received their basic training. Consistent with this, a study in Moldova found that participants rated public health activities significantly lower in importance than all other aspects of professional practice assessed (e.g. dispensing activities) [65]. Furthermore, a survey in Scotland offering participants a choice of hypothetical jobs found that participants would rather provide a minor illness service than health promotion advice and would forgo £2798 of income to do this [72].

Perceptions regarding the pharmacists' role in smoking cessation counselling were generally favourable. Nearly all pharmacists surveyed in Thailand, Finland and the USA agreed that they should play a role in smoking cessation [45,37,16]. The majority of participants (83%) in another survey in the USA believed that pharmacists should be more active in assisting with smoking cessation [55]. However, in a survey in Turkey only 57% of participants thought that pharmacists should warn patients about the harmful effects of smoking [50]. A study in Canada found that pharmacists rated medicine related aspects (e.g. advising on the use of NRT) of their smoking cessation role as more important than other aspects (e.g. assessing patients' dependence on nicotine) [34]. Another paper based on the same sample found that participants were significantly more likely to carry out smoking cessation interventions with customers if they scored above the median in ratings of importance of various smoking cessation roles [32].

Perceptions about the pharmacist's role in sexual health services were generally positive. The majority of pharmacists (98%) surveyed in a study in Scotland agreed that they would be willing to offer free Chlamydia postal testing kits [26]. In a survey in the USA 55% of pharmacists were interested in providing emergency hormonal contraception (EHC) [68]. Pharmacists interviewed in a study in the UK [69] were found to hold largely positive views about providing EHC. However, around one quarter of pharmacists in another study in the USA were opposed to providing EHC largely due to religious and moral beliefs [67]. Pharmacists in the latter two studies also reported concerns that the service may be overused and lead to increases in unprotected sex and sexually transmitted diseases [67,69]. The benefits of providing this service that were highlighted by pharmacists in these two studies included increasing access to EHC, confidentiality, reducing unwanted pregnancies and improving status of the pharmacy profession [67,69].

Attitudes towards providing services for drug misusers have become more favourable over recent years. Surveys in England and Scotland in 2007 reported that attitudes were significantly more positive since assessed in a similar survey in 1995 [38,60]. Similarly, a study in the USA reported an increase in the number of pharmacists who agreed that sterile needles should be made available through community pharmacy [39].

Despite a shift in attitudes, views towards providing services for drug misusers are still mixed. Pharmacists taking part in focus groups in Estonia and a survey in the USA highlighted a number of concerns about the effect of selling sterile needles on customers and business [58,41]. Support staff in a survey in Scotland also reported similar concerns [30]. Only half of support staff (52.6%) in this study thought that their pharmacy should provide services to drug misusers [31]. The possibility of providing free injecting equipment to drug misusers was met with strong resistance in the study in Estonia. The majority of pharmacists in a survey in Scotland disagreed or neither agreed or disagreed that HIV/hepatitis prevention is an important role for pharmacists [73].

However pharmacists supplying sterile needles for purchase in the USA and UK reported few problems providing this service and little detrimental effect on customers or their business [41,42]. Pharmacists selling sterile injecting equipment in Vietnam reported that they felt a responsibility to prevent blood borne infection and were willing to provide health education to customers that were drug misusers [43]. More positive views were also reported in a study in the USA with nearly all pharmacists (98%) reporting that they felt they should play a part in helping prevent the spread of blood borne infections such as HIV and over two thirds supporting the availability of sterile needles for purchase in community pharmacies [41].

#### Competence/Confidence

Findings regarding confidence and competence in providing health promotion services were mixed. A survey of pharmacists in Scotland found that around one third of participants did not feel that they were competent in promoting and protecting the populations' health or encouraging behavioural change [21]. Around two thirds felt they lacked the underpinning knowledge and one third felt they could not apply their knowledge. Pharmacists taking part in a survey in Moldova rated their competence in health promotion activities at between 2.9 and 3.6 (0 = low competence and 5 = high competence) which was lower than competence scores for all other aspects of professional practice [65]. In contrast, the majority of pharmacists (95%) in a survey in Nigeria felt confident in advising patients on health promotion [71].

Pharmacists in Australia were reasonably confident in providing a smoking cessation service, with a mean confidence score of 3.7 (1 = not confident and 5 = extremely confident), and did not report confidence as a major barrier to smoking cessation activity [19]. Nearly all participants (92%) in a study in Canada [33] agreed that pharmacists can be effective in promoting smoking cessation with most customers. In another study in the USA around two thirds of pharmacists thought that the effectiveness of pharmacist counselling was average or good [55]. In two of these studies confidence was found to be the greatest predictor of the amount of smoking cessation activity reported and in one perceived effectiveness was also a significant predictor [19,55].

Confidence in advising on the prevention HIV/hepatitis was fairly low in pharmacists in a survey in Scotland [73]. Around half of pharmacists felt confident in advising customers on prevention of HIV and around a third on Hepatitis B/C. Confidence in advising on safer sex was higher with the majority of pharmacists reporting that they would be able to give advice on this to any customer or a drug misuser (78% and 72% respectively). However, only around one third were confident in advising a gay man on safer sex (35%) [73]. Support staff in a similar survey in Scotland reported lower confidence for advising on safer sex than pharmacists [74]. Only half of support staff felt able to give accurate advice to any customer (51%) and one third a drug misuser (34%) or a gay man.

Pharmacists' confidence in achieving positive outcomes in weight management counselling was low in one study. Pharmacists in a study in the USA reported mean confidence (1 = not at all confident and 5 = extremely confident) scores of only 3.0 for achieving weight loss in patients as a result of pharmacist counselling and 2.8 for achieving consumption of a calorie controlled diet in patients [18]. Mean confidence scores for medicine related aspects of obesity counselling (e.g. minimisation of adverse effects of anti-obesity medication) were higher at between 3.3 and 3.4. Self reported frequency of obesity counselling was found to be positively correlated with confidence in achieving positive outcomes. Confidence in providing brief alcohol screening and interventions was also low with over half of pharmacists in a study in New Zealand feeling neutral or disagreeing that they could appropriately advise patients about drinking [61].

#### Barriers

A number of common barriers to public health practice were highlighted across the different services. These included availability of a private counselling area, time, customer demand/reaction and reimbursement for public health services.

The findings regarding a lack of private counselling area were mixed. This was identified as a main barrier to providing advice on health promotion in focus groups in Sweden and advice on prevention of HIV/hepatitis in pharmacists and support staff in Scotland [56,73,74]. Nearly two thirds of pharmacists in a survey in Canada felt that having a designated space in pharmacy was very or somewhat important in facilitating smoking cessation practice and nearly half of participants in a study in Thailand (43%) thought the pharmacy setting was barrier to smoking cessation counselling [35,45]. Pharmacists' perception of having adequate facilities was found to be a significant predictor of frequency of smoking cessation counselling in one study [55]. Although a predictor of service provision, the majority of pharmacists (71.7%) in this study did not view the pharmacy setting was an important barrier to smoking cessation counselling. Similarly, pharmacists in Nigeria (93.1%) did not think facilities were a barrier to patient interaction in relation to health promotion generally [71]. Pharmacists interviewed in England also felt they had adequate facilities to provide a Chlamydia testing and treatment service [22].

Lack of time was identified as a main barrier to providing advice on prevention of HIV/hepatitis by pharmacists and support staff in Scotland and for health promotion activities by the majority (75%) of pharmacists in a study in Malaysia [73-75]. Between 50 and 70% of participants in two studies in the USA and one in Thailand agreed that time was a barrier to providing smoking cessation counselling and over half in one of the USA studies felt that they were not adequately staffed for providing smoking cessation services [15,55,45]. Similarly, around 70% of participants in a study in New Zealand thought that being too busy was a barrier to carrying out brief alcohol screening [61]. Time was reported as a barrier to providing EHC by 67% of pharmacists surveyed in a study in the USA [67]. However, a study on health promotion in Nigeria and another on Chlamydia testing in England reported that time was not an issue in providing these services [71,22].

Views on patient demand for public health services in community pharmacy and patient reactions to being offered these services were mixed. Around 60% of pharmacists in a survey in Thailand reported that lack of patient demand was a barrier to providing smoking cessation services [45]. Focus group participants in Sweden also perceived that patients had low expectations of receiving health promotion advice from pharmacists [56]. Furthermore, over half of pharmacy assistants in a survey in Scotland felt that client embarrassment was a barrier to offering advice on HIV/hepatitis prevention and a similar proportion of pharmacists in a study in New Zealand felt that patients would resent being asked about their alcohol consumption [74,61]. Pharmacists interviewed in the USA reported that they viewed this as a sensitive topic and were hesitant to initiate conversations about smoking as they expected to receive a negative response from customers [62].

In contrast, the majority of participants in surveys on health promotion and smoking cessation in Nigeria, the USA and Finland did not think that lack of demand was a barrier and thought that patients were motivated to seek health advice from pharmacists, welcomed and valued this advice and were not discouraged from returning to the pharmacy as a result of being offered advice [71,33,55,37]. Pharmacists in a survey carried out in the USA agreed that customers are becoming more willing to discuss health problems and more accepting of counselling provided by pharmacists, but did not agree as strongly that customers were more accepting of pharmacists managing chronic disease [76]. Finally, pharmacists involved in offering a Chlamydia testing service reported that client reactions were to being offered the service were mixed but that they were predominantly satisfied as long as discretion was used [22].

Reimbursement for providing public health services does not seem to be a barrier for most pharmacists. The majority of participants (63.7%) in a study in Malaysia felt neutral or disagreed that a lack of reimbursement was a barrier to their involvement in health promotion and most pharmacists (87.6%) in a survey in Nigeria agreed that it is alright to be involved in health promotion whether there is reimbursement or not [75,71]. Studies in the USA, Thailand, and Canada also reported similar findings in relation to smoking cessation [55,45,33,34].

#### Training

A need for training was identified in a number of surveys on different public health services. Over half of pharmacists in a study in Scotland reported that attaining additional pharmaceutical public health knowledge was a priority for their practice now and two thirds thought it would be a priority in the future [21]. Between one third and one half of pharmacists in three studies felt that lack of training or lack of knowledge and skills was a barrier to their smoking cessation practice [15,50,55]. Pharmacists and support staff in Scotland also felt that lack of training was a main difficulty in providing advice on prevention of HIV/hepatitis and over 80% of pharmacists in a study in New Zealand felt it was a barrier to providing alcohol screening and brief interventions [73,74,61]. Over 70% of pharmacists in a survey in Scotland reported that they would like further training on drug misuse [59]. The majority of pharmacists in Nigeria felt that they had good knowledge on health promotion (86.9%) but also agreed that they would be willing to retrain on health promotion (93.2%) [71].

Pharmacists taking part in a smoking cessation training needs assessment in Canada reported that training would be helpful on all aspects of smoking cessation practice but rated training on behavioural techniques for quitting smoking and motivating patients as most helpful [34]. Pharmacists in Scotland taking part in a survey on training needs for working with drug misusers most often cited motivational and counselling skills as areas they would like more training on [77]. No clear area for future training was identified in a survey in Scotland with the majority of pharmacists agreeing (79.3%) that training should focus on generic knowledge and skills but also with the statement that training should focus on priority health issues such as chronic heart disease (77.2%) [21]. Training for pharmacy technicians on smoking cessation was found to significantly increase knowledge, confidence and perceptions of the effectiveness of smoking cessation counselling in a study in the USA [24].

### Consumers

The attitudes and beliefs of the general public and pharmacy customers towards pharmaceutical public health investigated in the papers in this review related to four topics: use of community pharmacies, appropriateness of pharmacists' involvement in public health, satisfaction with pharmaceutical public health and perceptions of pharmacists' ability.

#### Use of community pharmacies

A survey of pharmacy customers in Australia found that the majority had never received advice on diet and exercise (88.2%) or on preventing health problems (65.1%) from a pharmacist [47]. The majority of smokers (57.8%) in the sample also reported having never received advice on smoking from a pharmacist.

Most pharmacy customers in a survey in Sweden expected to receive information from pharmacists on drugs (80.5%), while only around a third (36%) expected information on general health issues and less than a quarter expected advice on diet (24%), smoking cessation (21%) or disease/illness (20.5%) [78]. Users of nicotine replacement therapy in a survey in the USA found it most useful and were most likely to discuss medicine related smoking cessation topics (e.g. side effects of smoking cessation medication) with a pharmacist and were least likely and found it least useful to discuss behavioural topics (e.g. how to cope with difficulties encountered) [52]. A survey of pharmacy customers in Nigeria found that satisfaction was lower for the availability of public health services than other medicine related services [20].

#### Appropriateness of pharmacists' involvement in public health

The majority of participants in studies on smoking cessation (83%), health screening and promotion (71% and 74% respectively), EHC (65%), services for drug misusers and Chlamydia testing (75%) thought that pharmacists were appropriate providers of these services [17,56,49,64,70]. Users of nicotine replacement therapy in a study in the USA on average rated the appropriateness of pharmacists taking an active role in smoking cessation as 6.9 out of 10 (1 = not at all appropriate and 10 = extremely appropriate) [52]. However, less than one quarter (22%) of participants surveyed at a medical centre thought that pharmacists should monitor long term conditions such as asthma [59].

#### Satisfaction with pharmaceutical public health

Although it seems that customers often do not expect or receive advice from pharmacists on public health topics, satisfaction in those that have experienced pharmacy public health services is high. A survey in Australia found significantly more positive attitudes in those that had experience of pharmacy health screening or promotion than those that did not [57]. Attitudes in those with no experience of public health services were also found to be significantly more positive compared to a similar survey carried out around seven years previously.

Nearly all individuals receiving community pharmacy osteoporosis screening and education in two surveys in the USA reported that the information provided increased awareness (98%), that they were satisfied with the interaction (92%) and found the advice valuable or highly valuable [79,53]. The majority of participants receiving self management interventions from community pharmacists for asthma (89%) and diabetes (97.5%) were also satisfied with the care they received from the pharmacist [54,80]. Only 71% and 61% of those receiving the asthma self management interventions were satisfied by the education and counselling provided by physicians and nurses respectively [54].

Participants in a survey in the USA reported very positive experiences of community pharmacy based smoking cessation services [40]. Patients' agreement with ten statements about their satisfaction with the service (1 = lowest satisfaction and 10 = highest satisfaction) was high with mean scores between 8.5 and 9.9 for all of the statements. Intravenous drug users taking part in focus groups in Estonia reported that pharmacies were more convenient and easier to access than other needle exchange services, but that they experienced discomfort and embarrassment as a result of perceived negative attitude of the pharmacist and other customers towards them [58].

Women who received EHC from community pharmacy reported largely positive experiences of this service. The majority of women participating in surveys, interviews and focus groups in the USA, Canada and England reported that they were satisfied with their consultation with the pharmacist [81,82,27,48,69]. Over 80% of women in the survey conducted in the USA and another in Canada were satisfied with the amount of privacy in the pharmacy [81,27]. The flexibility and convenience of the pharmacy setting were viewed as benefits to this setting and were the primary reason for attending pharmacy over than other settings such as family planning clinics [63,82,69]. Indeed the majority of women (65%) sampled for a study in Sweden reported that they would prefer to purchase EHC from a pharmacy over visiting a clinic with availability selected as the motive for this choice by most women (64%) [66].

Despite largely positive views towards the service, concerns were reported by women in some studies. Some participants in focus groups in Europe, interviews in England and interviews in the UK felt that there were issues with privacy in the community pharmacy setting [51,48,63]. Significantly more women who obtained EHC from other services (e.g. family planning clinics) in the survey in England reported that they felt comfortable, had adequate privacy, adequate advice, and had discussed future contraception than those attending pharmacy [48]. Participants in the focus groups in Europe and Sweden also expressed mixed views on their interaction with the pharmacists [51,25]. Some participants perceived that the pharmacist was judgemental towards them in the consultation [51,25].

The majority of women surveyed after taking a postal Chlamydia testing kit from a pharmacy in Amsterdam reported that it was a good method of screening (68%) [23]. In a similar study in England, the majority of customers taking a Chlamydia testing kit were very satisfied with the service (80%), found the consultation sufficiently private (95%) and were comfortable discussing sexual health with the pharmacist (100%) [70]. In telephone interviews participants commented on the excellent communication skills of the pharmacist and the short waiting times and anonymity at the pharmacy. However, the interviews also revealed that while customers were satisfied with the confidentiality of the consultation, there were concerns regarding confidentiality at the counter [70].

#### Perceptions of pharmacists' ability

Around one third of pharmacy customers in a survey in the UK were unsure if the pharmacist was qualified to issue advice on sexual health issues or had enough experience or knowledge to deal with sexual health related issues [44]. Approximately three quarters of patients surveyed at a medical centre in the USA were undecided, disagreed or strongly disagreed with the statement that pharmacists are trained to provide smoking cessation services [17]. Those that reported a greater frequency of discussing medications with their pharmacists were more likely to agree or strongly agree with the statement. In a sample of the general public in the USA, 82% and 94.2% of participants respectively thought that pharmacists and physicians would be a very good or somewhat good source of advice on quitting smoking [36]. Nicotine replacement therapy users in a survey in the USA rated pharmacists' smoking cessation knowledge as highest in relation to prescription medicines at an average of 8.1 out of a maximum score of 10 and lowest in relation to knowledge of non-drug strategies to help tobacco users to quit at an average of 4.0 [52].

Patients with type 2 diabetes in a study in the United Arab Emirates showed a significant increase in their perceptions of pharmacists' ability to help them to reduce their blood sugar after receiving a pharmacist led self management intervention [46]. At baseline 32% of participants agreed or strongly agreed that their pharmacist can help decrease their blood sugar and 92% agreed or strongly agreed with the same statement for their doctor. Over half of participants (56%) agreed or strongly agreed that the pharmacist could help after receiving the intervention. A survey in Sweden found that around three quarters of pharmacy customers thought that pharmacy could influence people's willingness to improve their health [78].

## Discussion

### Pharmacy staff

The majority of pharmacists in the review were positive about providing public health services and felt that this was an important role. This suggests that the changing role of community pharmacy from traditional dispensing activities to greater involvement in health improvement is largely accepted, and the importance of providing these services is understood. However, the review indicates that the public health role is still considered secondary to medicine related roles. Pharmacists viewed public health activities as less important than traditional roles and were less confident in providing these. Less positive views were also held by some pharmacists in relation to certain public health services, particularly services for drug misusers. These findings are consistent with those of the previous systematic reviews on this topic [6,7,12].

Reported levels of confidence in providing public health services varied from service to service in the current review, but on the whole were average to low. Confidence in our ability to perform a behaviour (known as self efficacy) has been found to be crucial in predicting whether we engage in the behaviour [83]. A review found that self efficacy can predict as much as 35% of the variance in behaviour [84]. This link between confidence and behaviour was supported in the current review with two studies reporting that confidence was the greatest predictor of self reported smoking cessation activity in pharmacists [19,55].

This review and the previous review identified a need for further training for a number of different public health services. Encouragingly, training was found to have a positive effect on pharmacists' attitudes in the previous reviews [6,7,10]. Although very few studies in the current review assessed specific areas for future training, the findings of the review do suggest how training may be best targeted. A study on drug misuse and another on smoking cessation indicated that training on motivating patients and behavioural techniques would be most useful [77,34]. Training for health professionals often devotes time to explaining the importance of the health issue in question and what part the professional can play in tackling this. However, the findings of this review suggest that pharmacists understand this and that tackling pharmacists' self efficacy may have a much greater impact on practice. Although self efficacy is an important determinant of behaviour, there is limited evidence on interventions to increase self efficacy [85]. Therefore, further research is needed to investigate interventions to increase pharmacists' self efficacy for providing public health services.

Although most studies found that a lack of patient demand and negative customer reactions were not a barrier to public health practice, some pharmacists did perceive these to be a problem. Similarly, the previous review found that pharmacists were concerned about being intrusive when offering public health services. These beliefs could be addressed during training by outlining the results of research on customer experiences of pharmaceutical public health.

Time was fairly consistently identified in the current review as a barrier to a number of public health services. Similarly, the previous review reported that dispensing duties were a barrier to public health activities. Findings in the current review were more mixed regarding availability of an adequate counselling space as a barrier to public health practice, which may reflect differences in availability of counselling areas rather than differences in perceptions of their importance. Remuneration for providing public health services was not reported to be a barrier in the current review suggesting that current levels of remuneration are perceived to be adequate.

Only three studies in the current review reported the attitudes of support staff separately from those of pharmacists [22,56,30]. Fewer support staff reported that they were confident in providing accurate advice on prevention of HIV and hepatitis [22] than pharmacists in another similar survey [18]. Support staff are often the first point of contact for pharmacy customers and can play a vital role in alleviating the time pressures on pharmacists by offering public health services and carrying out initial screening. Research is needed to establish the attitudes of support staff to allow support and training to be appropriately targeted for this group.

### Consumers

Customer attitudes towards pharmaceutical public health were on the whole quite positive. Customers found the pharmacy a convenient setting and felt that pharmacists should provide public health services. Those that had experienced public health services in community pharmacy, such as self management interventions, emergency hormonal contraception and Chlamydia screening, were largely satisfied with their experience of these. However, two studies revealed that most customers did not expect, and had never been offered, public health advice from a pharmacist [47,78]. This suggests that pharmacists' perceptions of low demand for public health services are accurate. However, the expectation of a negative customer reaction to pharmaceutical public health services held by some pharmacists seems to be unfounded. These findings are also consistent with those of the previous systematic reviews in the area [6,7,12]. Customers in the previous review valued the pharmacists input in public health services, but perceived pharmacists as drug experts and did not often use pharmacies for general health advice.

Customers' perceptions of pharmacists' ability to provide public health services were mixed in the current review, with some perceiving pharmacists as good sources of advice on health and other not. One study found a significant increase in customers' perceptions of pharmacists' ability after receiving a diabetes self management intervention from pharmacist. Similarly another study reported significantly more positive attitudes in members of the public who had experienced pharmacy health screening or promotion than those that had not.

Issues with privacy were raised in four studies investigating sexual health services in community pharmacy and were also highlighted in the previous review on customer attitudes [48,51,63,70,11]. Although private counselling areas are becoming more common in pharmacy, these studies highlight that there are still issues with privacy, particularly at the pharmacy counter. The nature of the pharmacy setting can make complete privacy difficult to achieve, but future projects involving sensitive topics such as sexual health must make attempts to remedy this issue.

Pharmaceutical public health services are clearly well received by those that experience them and result in high levels of satisfaction. Considering the findings of this review, it seems likely that the more these services are offered and experienced by the general public, the more positive attitudes will become. Successfully changing the public's perception of pharmacist in this way will require pharmacists to be proactive in offering public health services. As discussed, pharmacists may need additional support and training in order to feel confident about doing this.

### Limitations of the review

Heterogeneity in the included studies reduced the ability of the review to summarise trends and may have accounted for some of the inconsistencies in findings. Studies were conducted in a variety of countries across the world with differing health care systems and it is not known how these different cultures and systems may affect customer and pharmacy staff attitudes. A number of factors increased the risk of bias within the included studies. Response rates were generally average, with the majority of studies achieving rates of 50% and over. One quarter of studies did not report response rates and only three studies followed up and assessed non-respondents. Convenience sampling was used in the majority of studies included in the review. These factors may mean that the views of those sampled were not representative of the population as a whole and therefore limit the generalisability of the findings.

## Conclusions

The consistency of the findings of the current review with the previous reviews is striking. Despite the introduction of public health services to the pharmacy contract in the UK, current attitudes of pharmacists and the public appear to be largely similar to before these changes. Although this is discouraging it is perhaps not surprising considering the magnitude of the changes pharmacists have experienced in their role. Around half of non-UK papers and one third of UK papers in the present review were published prior to the introduction of the new pharmacy contract in the UK (See table 2). This fact considered with the possible lag between research being conducted and published may also partly explain why there was little difference found in attitudes between the current and previous systematic reviews.

It is important that the positive attitudes of pharmaceutical public health users and pharmacists found in both reviews are extended and built upon. Appropriate training and support is needed in order increasing pharmacists' confidence in providing public health services. Future research needs to investigate the effectiveness of strategies for increasing pharmacists' confidence and changing their public health practice. If pharmacists can be supported to offer public health services more proactively, it is likely that increased exposure to public health services will have a positive effect of the attitudes and health of the general public.

## Competing interests

The authors declare that they have no competing interests.

## Authors' contributions

CE conducted the design, screening, data abstraction, data analysis, and drafted the manuscript. JF assisted in the design of the study and helped draft the manuscript. RO assisted in the design of the study, assessing papers for inclusion and drafting the manuscript. All authors read and approved the final manuscript.

## Pre-publication history

The pre-publication history for this paper can be accessed here:

http://www.biomedcentral.com/1471-2458/11/582/prepub

## Supplementary Material

Additional file 1 **Characteristics of included studies**. Excel table showing characteristics of studies included in the review. Click here for file
